# Event and Comment

**Published:** 1914-01-15

**Authors:** 


### DENTAL REGISTER.

**Vol. LXVIII. January 15, 1914. No. 1**

### EVENT AND COMMENT.

DENTAL BRIEF DISCONTINUES PUBLICATION. With the December issue the *Dental Brief* which has been published by the L. D. Caulk Co., ceased publication. This will come as an unwelcome surprise to many who have come to believe that this journal was one of our permanent and most useful literary organs. The publishers give as their reasons the fact that they are compelled to exclude from their business cares everything that in any way interferes with the conduct of their first business of the manufacture and sale of dental supplies. This is a frank and very proper statement as reached from the business man's viewpoint; but the profession has got into the habit of expecting our supply houses to furnish it with intellectual as well as material supplies, and there, no doubt, will be a general demur when so excellent a journal as the *Brief* is no longer available. Under the able editorial care of the late Dr. Litch and his associates Drs. Thorpe and Trueman, this journal has made a place of usefulness for itself which no other journal has occupied. We often wonder if the hard and faithful work which such men put into their efforts to keep before the profession, the highest standards of professional progress is properly appreciated, and why the profession in the mass takes so little interest in its literature, either in its making or in giving it a reasonable consideration. It may be answered that it is not as worthy as it should be; but this is no answer, as every one who thinks at all must know that the profession and not the editors or publishers in a large measure make, or at least inspire, the production. We are sorry to loose the *Brief*, but we hope the new journal of the National Society may take its place.

OHIO STATE ANNUAL MEETING. The forty-eighth annual meeting of this society was held in Toledo, December 2nd, 3rd and 4th. Because of the efforts of the local committee the arrangements were all that was to be expected, and the business and entertainment affairs were conducted with rare skill and eminent satisfaction to all the visitors. The hotels were taxed to their limits to entertain the crowd, but they proved themselves equal to the occasion.

The program was unusual, it was not an Ohio man program, but every section of the country from Atlanta to Minneapolis was represented by their particular stars. In some respects the meeting may have been mistaken for a meeting of the National Dental Association, with the Governor of Ohio and the President of the American Medical Association as special guests.

Dr. F. T. Van Woert, of Brooklyn, N. Y., opened the literary program by presenting as a sterioptican lecture his method of securing impressions for inlay work, as described by him in the *Items of Interest* during the past year, in a series of articles beginning January, 1913. Dr. Van Woert is an enthusiast in his work, a man of great technical skill and has a scientific habit of investigating and classifying technical matters which makes his work conclusive. His lecture was ably discussed and amplified by Dr. J. V. Conzett, of Iowa, E. B. Spalding, of Detroit, Karl G. Knocke, of Chicago, and A. E. Schneider, of Chicago, in written discussions. It was certainly a rare treat and we do not know how the subject could have been presented in a more conclusive manner. This material when printed will go into our scrap book.

An innovation that will be hard to duplicate was the "President's Night." Instead of the usual President's address, Dr. Price gave what every one said was a most inspiring and enlightening lecture on the results of some scientific researches he has been making on a variety of subjects: silicate cements, shrinkage of casting metals, Infant foods; an electric apparatus for registering the temperature, respiration and heart beat of patients in hospitals. This is a most ingenious affair, and dispenses with the constant supervision of the bedside nurse, as it is so active and vigilant that it records all variations accurately, and should a dangerous condition develop a bell is rung in the office of the nurse in an adjoining room or the physician miles away if necessary. The same machine can be applied to all the patients of an entire ward as well as to a single individual, so that the physical changes in every patient can be a matter of definite record. The address was not only unusual for its material, but was delivered with such clear diction and in such an enthusiastic manner, that every one was charmed and convinced of its great value to both the dental and medical professions. Dr. E. C. Kirk, who discussed the address said it was like attempting to discuss the new edition of the encyclopedia Brittanica, and Dr. V. C. Vaughn, President of the American Medical Association, said it was a most valuable contribution to medical as well as dental science. It is remarkable that a busy practitioner can find time to do such work; but we find that where the spirit is there is certain to be more or less activity, and Dr. Price seems not only possessed of the spirit, but the determination also that compels time and produces results.

Wednesday and Thursday forenoons were given over entirely to clinics. The men engaged and the method adopted to permit every spectator to see every clinic was admirably carried out. Twenty clinics were given and each by a well known specialist. Van Woert on inlays; Conzett, Cavity Preparation; Wilson, Vulcanizing Rubber; Bush, Removable Bridge Work; Ladd, Serum Treatment Pyorrhea; Harris, Bacterial Treatment Pyorrhea; Hartzell, Pyorrhea Instrumentation; Weaver, Antral Diseases; Skinner, Borated Oxygen for Pyorrhea; Jackman, High Pressure Anesthesia; W. I. Jones, Nitrous Oxide and Oxygen Anesthesia; G. N. Wasser, Nitrous Oxide and Oxygen Analgesia; M. L. Ward, Inlay Cavity Preparation; A. L. LeGro, Porcelain Jacket Crown; K. G. Knocke, Crown and Bridge Work; J. L. Kelly, Removable Bridge Work; A. E. Schneider, Porcelain Crowns; J. M. Thompson, Gold versus Cast Fillings; F. L. Olds, Cast Metal Plates; J. H. Prothero, Accurate Bite and Transfer Method.

The method of conducting the clinics was by what has been termed the progressive plan. Each clinician is numbered and he is given a space far enough from his neighbors not to be interrupted or confused by other clinics. The audience is divided into groups of equal numbers and are assigned a letter of the alphabet and are required to stay with their banner and visit the clinicians in a regular order. Each clinician was allowed fifteen minutes to present his clinic which he did to the several groups of spectators in a regular sequence. By this means the clinicians presented his clinic in a concise form without interruptions of any kind to every person. From the spectators standpoint the scheme is excellent, but it is exceedingly tiresome to the clinician. For some clinics the time was too short to do full justice, and there was no time for the audience to ask questions, which perhaps is not an adverse criticism, as in a miscellaneous crowd many foolish questions are a source of annoyance and delay. We would suggest that some modification of the plan whereby some clinics could have more time and others less would be helpful, so that the groups would not be unoccupied some of the time, and also that some scheme by which clinicians who have to talk so much could have an occasional rest period. We would suggest that thirty minute periods be allowed each group, and that two clinicians be assigned where the clinics can be made in fifteen minute periods, so that each clinician may have a rest period of fifteen minutes; and for clinics requiring a longer time that one clinician be assigned to a thirty minute period, such clinician can prepare his clinic in such a way as to give it, and do at least his talking, in say twenty minutes, and the other ten minutes be given over to the inspection of models, patients, or to a questionaire, which may be in charge of an assistant to the clinician. Some such scheme would accomplish the same object and overcome some of the objections where the present plan is carried out. In this way every minute of the time would be utilized and nobody need be overworked. There is another objection which is of some importance to this method, and it is that the number of clinics that can be given at any ordinary meeting must be confined to the time at the disposal of the meeting, and if the clinic committee selects an all-star program, some of the worthy or modest men may never have a chance to present their ideas; and there will be no incentive for the young man of moderate ability to engage in the usual forms of society work. Are we in danger of fostering a clinical aristocracy, or of impoverishing our future developments by depriving our younger and unknown men of a chance to develop under the inspiring influences of a public exploitation of their conceptions and works?

The program for Wednesday afternoon was a symposium of two papers on oral diseases. One by Dr. C. J. Grieves, on peridental infections of an unusual character accompanying a throat disease that was epidemic in Baltimore, caused by an impure milk supply. The other by Dr. Hartzell on the pathology and up-to-date treatment of Pyorrhea. Both papers were exhaustive and of great interest and value, and when published should be studied with care by every one interested in any form of mouth infection.

One of the most delightful and interesting features of the program was the evening given over to a banquet at the Toledo Commercial Club. After dinner addresses were made by Dr. H. C. Brown, President of the National Dental Association; Governor Cox, by his private secretary; Dr. V. C. Vaughn, President American Medical Association; Dr. E. C. Kirk, Editor *Dental Cosmos;* and Dr. E. F. Mc Campbell, Secretary of Ohio State Health Board. Every one of these addresses was a gem of its kind. It was the general concensus of remark from the audience that it was hardly to be expected that so much real wisdom of so varied a kind —not to mention the keen wit of the various speakers— would ever again be presented at a dental society banquet. We are of the opinion that few men would have had the foresight to present such a galaxy of after dinner speakers on such varied and yet allied subjects, or have the tact to appropriately present them to the audience with introductory remarks that not only prejudiced the audience, but compelled each speaker to his best efforts.

It was a grand meeting, and Dr. Price and his associates in office deserve the honor of preparing and successfully carrying out a most remarkable program, which future officers can scarcely expect to surpass. It was really an exceptional gathering of brilliant men.

